# Risk factors for post-acute sequelae of COVID-19 in hospitalized patients: An observational study based on a survey in a tertiary care center in Türkiye

**DOI:** 10.5578/tt.20239707

**Published:** 2023-09-22

**Authors:** Y. Yıldız, B. Öztürk Şahin, M.C. Taşdemir, Ş. Demir, B. Çifci, N. Köktürk, T. Ulukavak Çiftçi, A.S. Yurdakul, N.Y. Demirci, M. Aydoğdu, M. Dizbay, İ.K. Oğuzülgen

**Affiliations:** 1 Department of Infectious Diseases and Clinical Microbiology, Gazi University Faculty of Medicine, Ankara, Türkiye; 2 Clinic of Pulmonary, Şehitkamil State Hospital, Gaziantep, Türkiye; 3 Department of Pulmonary, Gazi University Faculty of Medicine, Ankara, Türkiye

**Keywords:** COVID-19, post-acute COVID-19 syndrome, risk factor, SARS-CoV-2 infection, long COVID, COVID-19, akut COVID-19 sonrası sekel, risk faktörü, SARS-CoV-2 enfeksiyonu, uzamış COVID

## Abstract

**ABSTRACT:**

Risk factors for post-acute sequelae of COVID-19 in hospitalized patients: An observational study based on a survey in a tertiary care center in Türkiye

**Introduction:**

Long COVID is a multisystem disease with various symptoms and risk factors. We aim to investigate the post-acute sequelae of COVID-19 and related risk factors in a tertiary care center.

**Materials and Methods:**

In this observational study, based on a survey of 1.977 COVID-19 patients hospitalized from April 2020 to January 2021, a retrospective assessment was carried out on 1.050 individuals who were reachable via telephone to determine their eligibility for meeting the inclusion criteria.

**Results:**

The data of 256 patients who reported at least one persistent symptom were analyzed. Long COVID prevalence was 24.3%. Among 256 patients (median age 52.8; 52.7% female; 56.63% had at least one comorbidity), dyspnea, fatigue, arthralgia-myalgia, cough, and back pain were the most common post-acute sequelae of COVID-19 (42.4%; 28.29%; 16.33%; 13.15% and 7.17%, respectively). The risk factors for the persistence of dyspnea included having lung diseases such as chronic obstructive pulmonary disease, a history of intensive care support, the requirement for long-term oxygen therapy, and a history of cytokine storm (p= 0.024, p= 0.026, p< 0.001, p= 0.036, p= 0.005, respectively). The correlation between lung involvement with post-discharge cough (p= 0.041) and dizziness (p= 0.038) was significant. No correlation between the symptoms with the severity of acute infection, age, and gender was found. When a multivariate regression analysis was conducted on the most common long COVID-related symptoms, several independent risk factors were identified. These included having lung disease for dyspnea (OR 5.81, 95% CI 1.08-31.07, p= 0.04); length of hospital stay for myalgia (OR 1.034, 95% CI 1.004-1.065, p= 0.024); and pulmonary involvement of over 50% during COVID-19 infection for cough (OR 3.793, 95% CI 1.184-12.147, p= 0.025).

**Conclusion:**

COVID-19 survivors will require significant healthcare services due to their prolonged symptoms. We hope that our findings will guide the management of these patients in clinical settings towards best practices.

## Abstract (tr)

**ÖZ:**

Hastanede yatan hastalarda akut COVID-19 sonrası sekeller için risk faktörleri: Türkiye’de üçüncü basamak bir sağlık merkezinde ankete dayalı gözlemsel bir çalışma

**Giriş:**

Uzamış COVID, çeşitli semptomları ve risk faktörleri olan bir çoklu sistem hastalığıdır. Bu çalışma ile üçüncü basamak bir merkezde COVID-19’un akut sonrası sekellerini ve ilgili risk faktörlerini araştırmayı amaçladık.

**Materyal ve Metod:**

Bir ankete dayanan bu gözlemsel çalışmada, 2020 Nisan’dan 2021 Ocak’a kadar hastanede yatarak takip edilen 1,977 COVID-19 hastasından telefonla ulaşılabilen 1,050’si dahil etme kriterlerine uygunluk açısından geriye dönük olarak değerlendirildi. Taburculuk sonrası en az sekiz hafta devam eden en az bir uzamış semptom bildiren 256 hastanın verileri analiz edildi.

**Bulgular:**

Uzamış COVID prevalansı %24,3 bulundu. 256 hasta arasında (medyan yaş 52,8; %52,7 kadın; %56,63’ünde en az bir komorbidite vardı); nefes darlığı, yorgunluk, artralji-kas ağrısı, öksürük ve sırt ağrısı COVID-19’un akut sonrası en yaygın sekelleriydi (sırasıyla %42,4; %28,29; %16,33; %13,15 ve %7,17). Kronik obstrüktif akciğer hastalığı başta olmak üzere akciğer hastalığı tanısı olmak, yoğun bakım desteği öyküsü, uzun süreli oksijen tedavisi ihtiyacı ve sitokin fırtınası öyküsü olmak, nefes darlığının devam etmesi için risk faktörleriydi (sırasıyla p= 0,024, p= 0,026, p< 0,001, p= 0,036, p= 0,005). Akciğer tutulumu ile taburculuk sonrası öksürük (p= 0,041) ve baş dönmesi (p= 0,038) arasındaki ilişki anlamlıydı. Semptomlar ile akut enfeksiyonun şiddeti, yaş ve cinsiyet arasında bir ilişki bulunmadı. Uzamış COVID ile en sık ilişkili semptomlar için çok değişkenli regresyon analizi yaptığımızda, akciğer hastalığına sahip olmanın (OR 5,81, %95 GA 1,08-31,07, p= 0,04) nefes darlığı; hastanede kalış süresinin (OR 1,034, %95 GA 1,004-1,065, p= 0.024) miyalji ve COVID-19 enfeksiyonu sırasında >%50 akciğer tutulumu olmasının (OR 3,793, %95 GA 1,184- 12,147, p= 0,025) öksürük için bağımsız risk faktörleri olduğunu saptadık.

**Sonuç:**

COVID-19 geçiren hastalar uzayan semptomları nedeniyle sağlık sistemlerinde önemli yük oluşturacaklardır. Bulgularımızın bu hastaların kliniklerde yönetiminde, en iyi uygulamalara rehberlik edeceğini umuyoruz.

## INTRODUCTION

Long COVID (post-acute sequelae of COVID-19; PASC) represents new symptoms that affect everyday function, emerge within four weeks to three months after first being infected, and last for at least two months. These symptoms may fluctuate over time and overlap with prolonged symptoms of post-hospitalization. The symptoms following acute COVID-19 are highly variable and encompass a wide range, including general symptoms (chronic fatigue, pain, poor exercise tolerance), respiratory symptoms (dyspnea, cough), cardiovascular symptoms (chest pain, palpitations), neurological symptoms (poor concentration, cognitive impairment, headache, sleep disturbance, dizziness, etc.), gastrointestinal symptoms (abdominal pain, nausea, vomiting, diarrhea, etc.), musculoskeletal symptoms (joint pain, muscle pain), ear, nose, and throat symptoms (tinnitus, loss of taste and/or smell, etc.), dermatological symptoms (skin rashes, hair loss), psychiatric symptoms (depression, anxiety, post-traumatic stress disorder), as well as renal, hepatic, endocrine manifestations, and thromboembolism ( 1 , 2 , 3 ).

The majority of individuals show improvement within six months after a COVID-19 infection. However, between ten to thirty percent of those who had COVID-19 reported experiencing at least one persistent symptom up to six months after the virus had cleared from their bodies. In a smaller subset, around 1-5% of all COVID-19 patients, the illness persists chronically, with an ongoing presence of one or more symptoms for more than six months. The estimated prevalence of long COVID is not yet known, as it varies across different countries ( 4 , 5 , 6 ).

There is preliminary evidence suggesting various pathways for the pathogenesis of long COVID. These include direct neuro-invasion, leading to symptoms like anosmia, olfactory and central nervous system manifestations; dysregulated immune responses and auto-inflammation; post-ICU syndrome contributing to prolonged pulmonary issues; and the development of pulmonary fibrosis. One question that arises is whether there exists any evidence of the persistent virus in immunologically privileged sites. Given that COVID-19 causes endothelial injury, the distribution of signs and symptoms could involve abnormalities in end organs due to ongoing endothelial dysfunction. Pathogenesis involves multiple causal factors, and various systems are impacted in distinct ways. Therefore, we need to evaluate multiple types of potential therapeutic interventions ( 7 ).

The scale and significance of this epidemic of long COVID necessitate its management by primary care providers within the primary care setting. The most effective approach is a patient-centered one, aiming to enhance individual quality of life and functionality. The primary principle to acknowledge is that this constitutes a legitimate clinical phenomenon, and one should not disregard the clinical signs and symptoms when confirming the diagnosis. We can reassure patients, provide mental health support, and manage some of the symptoms. It is equally crucial to understand that if laboratory values and imaging do not reveal abnormalities, these assessments do not solely define a patient’s overall well-being. A lack of abnormalities does not invalidate a patient’s symptoms. CDC advises a conservative diagnostic approach which will allow most cases to resolve. Setting achievable goals via shared decision-making will ameliorate specific symptoms and improve physical, mental, and social well-being ( 88 ).

To establish a clearer understanding of the post-acute sequelae of SARS-CoV-2 (PASC) and the factors that predict the persistence of symptoms following recovery from acute SARS-CoV-2 infection, we conducted a retrospective data collection from a cohort of 1.977 COVID-19 inpatients.

## MATERIALS and METHODS

## Study Design

This observational study was conducted on patients, who were diagnosed with laboratory-confirmed SARS-CoV-2 infection and hospitalized from April 2020 to January 2021 at a tertiary care center in Türkiye.

## Inclusion and Exclusion Criteria

Patients with SARS-CoV-2 PCR test positivity in the nasopharynx and/or lower respiratory tract samples who were hospitalized in COVID-19 wards and followed for more than 24 hours were investigated. Patients who were contacted by telephone and had at least one symptom that persisted for at least eight weeks after discharge were included in the study. Patients under 18, outpatients, and those needing intensive care support at hospital admission were excluded.

## Ethical Statement

All procedures undertaken in the study involving human participants adhered to the ethical standards set by the institutional and national research committees, in accordance with the 1964 Helsinki Declaration and its subsequent amendments, or equivalent ethical standards. Approval was granted by the Clinical Research Ethics Committee (Date 26.04.2021, No E.80610) and from the Turkish Ministry of Health Clinical Research Platform (No: 2020-05-01T21_53_43).

## Consent to Participate

Informed consent was obtained from all individual participants included in the study.

## Data Collection

Between April 2020 and January 2021, a total of 1.977 patients were hospitalized in isolation services. 343 patients died during hospitalization. Out of the 1.634 patients who were discharged, 1.112 patients were successfully contacted via phone. Among these, it was established that 42 of the 1.112 patients had died, and 20 patients were still hospitalized due to various reasons following their discharge.

**Figure 1:**
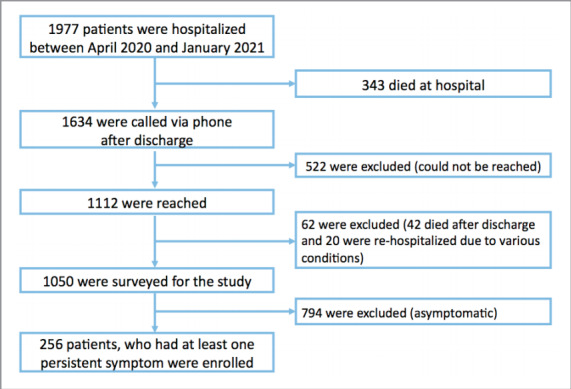
Flowchart of the study

A total of 1.050 patients were interviewed via phone using questionnaires specifically designed for this study. Among them, 256 patients who reported the persistence of at least one symptom after discharge were included in the analysis. The flowchart of the study is shown in Figure 1

The database was established, encompassing a range of information such as age, gender, chronic lung disease, other comorbidities, initial symptoms (fever, cough, dyspnea, etc.), and laboratory parameters recorded on the first day of hospitalization (D-dimer, troponin, ferritin, procalcitonin, IL6, CRP, white blood cell and lymphocyte count, creatinine, BUN, AST, ALT, LDH, bilirubin, sodium). Additional data collected included oxygen requirement, extent of lung involvement during the hospital stay, COVID-19 treatment received (antiviral, anticoagulant, or anti-inflammatory drug usage), planned treatment upon discharge, and ongoing symptoms during the course of long COVID for all 256 patients. This information was gathered by the researchers through telephone interviews and retrieval from the hospital information management system.

## Definitions

COVID-19 infection is defined as a positive SARS-CoV-2 molecular test result via nasopharyngeal/ oropharyngeal swab. Asymptomatic, mild, moderate, severe, and critical COVID-19 infections were determined according to the National Institute of Health (NIH) COVID-19 treatment guidelines ( 9 ).

Asymptomatic infection: Individuals who test positive for SARS-CoV-2 using a virologic test [i.e., a nucleic acid amplification test (NAAT) or an antigen test] but do not exhibit any symptoms consistent with COVID-19.

Mild illness: Individuals who have any of the various signs and symptoms of COVID-19 (e.g., fever, cough, sore throat, malaise, headache, muscle pain, nausea, vomiting, diarrhea, loss of taste and smell) but do not have shortness of breath, dyspnea, or abnormal chest imaging.

Moderate illness: Individuals who exhibit signs of lower respiratory disease upon clinical assessment or imaging and maintain an oxygen saturation level measured by pulse oximetry (SpO2) of 94% or higher while breathing room air at sea level.

Severe illness: Individuals who have SpO2< 94% on room air at sea level, a ratio of arterial partial pressure of oxygen to fraction of inspired oxygen (PaO2/FiO2) <300 mm Hg, a respiratory rate >30 breaths/min, or lung infiltrates >50%.

Critical illness: Individuals who have respiratory failure, septic shock, and/or multiple organ dysfunction.

Cytokine storm is defined as a variety of conditions that may ultimately result in pulmonary damage, acute respiratory distress syndrome (ARDS), multi-organ failure, and death due to uncontrolled immune system response and excessive release of cytokines, irrespective of the viral load present ( 10 , 11 ). Long COVID was defined according to relevant guidelines ( 1 , 12 ).

## Outcome Measures

The primary objective of the study was to identify the post-acute sequelae symptoms experienced by hospitalized patients, persisting for a minimum of eight weeks following the diagnosis of acute SARS-CoV-2 infection. The secondary goal of the study was to ascertain the associated risk factors.

## Statistical Analysis

All data were collected and analyzed by IBM SPSS Statistics version 26.0 (IBM Corp., Armonk, N.Y., USA). The Shapiro-Wilk test, histogram, and Q-Q plots determined the normality of the data distribution. The categorical variables were expressed as frequency and percentage, and the association among categorical variables was analyzed using either Pearson’s Chi-square test or Fisher’s exact test. The continuous variables were represented as a mean and standard deviation (SD) or median and first and third quartiles (Q1-Q3). The comparisons of two independent variables were examined with two samples t-test or Mann-Whitney U test based on the normality assumption. To determine risk factors for PASC, multivariate models including parameters with a p-value of <0.2 in univariate analysis of demographical characteristics and clinical findings were performed ( 13 ). p< 0.05 was considered statistically significant.

## Power Analysis

In the meta-analysis by Chen et al., the global prevalence of long COVID was determined to be 43%. In our study, the prevalence of long COVID was 24.3%. For a sample size of 1.050, the power of our study with 0.01 type 1 error is 100% ( 14 ).

## Data Availability

The data associated with the paper are not publicly available but are available from the corresponding author upon reasonable request.

## RESULTS

A total of 256 patients with at least one long COVID-related symptom were included in the study. Demographic characteristics and clinical findings are presented in Table 1 .

**Figure 2:**
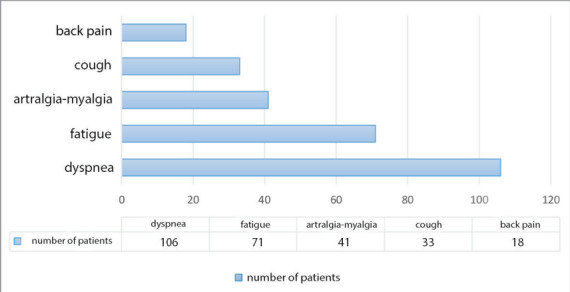
The most common five post-acute sequelae of COVID-19 (PASC) according to the number of patients (n= 256)

We identified the five most common post-acute sequelae of SARS-CoV-2 (PASC) and their duration, persisting for a minimum of eight weeks after discharge, as follows, dyspnea (42.40%; lasting from seven to 300 days), fatigue (28.29%; lasting from 10 to 300 days), arthralgia-myalgia (16.33%; lasting from two to 270 days), cough (13.15%; lasting from 10 to 360 days), and back pain (7.17%; lasting from 15 to 180 days). The long COVID-related symptoms of the 256 patients are displayed in Table 2 and Figure 2 , categorized by their frequency and duration.

Univariate analyses were performed for each PASC symptom to assess the correlation between demographic characteristics and clinical findings. Only the results that were found significant are summarized under the sub-headings dyspnea, cough, loss of smell, and dizziness.

## Dyspnea

Among patients with known lung disease, the symptom of dyspnea was more frequently reported as a PASC compared to other symptoms, and this difference was statistically significant. Shortness of breath was observed in 60% of those with lung disease and 40.6% of those without (p= 0.024).

Dyspnea was observed in 66.67% of the patients with a history of hospitalization in the intensive care unit and in 39.7% of the patients who were not admitted to the intensive care unit (p= <0.001). Dyspnea was observed in 63.64% of patients discharged with oxygen therapy and 40.5% of those who did not require oxygen therapy (p= 0.036). A statistically significant correlation exists between cytokine storm and dyspnea (p= 0.005). Dyspnea was observed in 63.9% of those with cytokine storm and 36.11% without (p= 0.005).

When examining the risk factors associated with the persistence of dyspnea eight weeks after the diagnosis of acute COVID-19 infection, significant associations were observed with known lung disease (including the presence of COPD), a history of intensive care support during the acute infection, the requirement for long-term oxygen therapy upon discharge, and the occurrence of a cytokine storm during hospitalization (p= 0.024, p= 0.026, p< 0.001, p= 0.036, p= 0.005 respectively).

## Cough

Post-discharge cough was observed in 12.41% of patients with pulmonary involvement of <50% and 25.64% with pulmonary involvement of >50% during COVID-19 infection (p= 0.041).

## Loss of Smell

A significant correlation was found between loss of smell and length of stay during COVID-19 infection (p= 0.038).

## Dizziness

A statistically significant correlation was found between lung involvement during COVID-19 infection and dizziness (p= 0.038). Dizziness was found in 10.3% of those with >50 involvement and 2.1% with <50 involvement.

## Other

No statistically significant relationship was found between any of the long COVID symptoms and age or gender. No correlation was found between chest pain, backache, headache, myalgia, malaise, and loss of taste and patients’ demographic characteristics and clinical findings.

**Figure 3:**
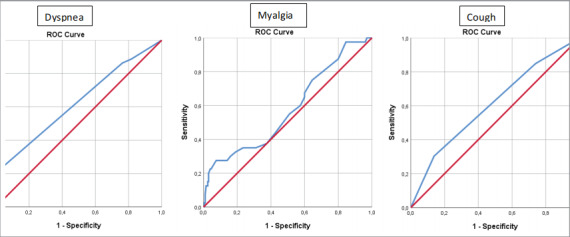
ROC curves of multivariate logistic regression analyses for the risk factors of dyspnea, myalgia, and cough as a PASC. Having lung disease (OR 5.81, 95% CI 1.08-31.07, p= 0.04) was found to be an independent risk factor for the development of dyspnea. The Hosmer-Lemeshow test for goodness of fit indicates that the model adequately fits the data (χ2= 3.006, p= 0.223). The area under the ROC curve with a 95% confidence interval for predicted probabilities was found as 0.622 (0.501-743) indicating poor discrimination. Length of hospital stay (OR 1.034, 95% CI 1.004-1.065, p= 0.024) was found to be an independent risk factor for myalgia. The Hosmer-Lemeshow test for goodness of fit indicates that the model adequately fits the data (χ2= 12.359, p= 0.136). The area under the ROC curve with a 95% confidence interval for predicted probabilities was found to be 0.578 (0.478-0.678), indicating poor discrimination. Pulmonary involvement of >50% during COVID-19 infection (OR 3.793, 95% CI 1.184-12.147, p= 0.025) was found to be an independent risk factor for cough. The Hosmer-Lemeshow test for goodness of fit indicates that the model adequately fits the data (X^2^< 0.001, p> 0.999). The area under the ROC curve with a 95% confidence interval for predicted probabilities was found to be 0.608 (0.502-0.714), indicating poor discrimination.

We conducted multivariate logistic regression analyses for common PASCs. For dyspnea, having lung disease (OR 5.81, 95% CI 1.08-31.07, p= 0.04); for myalgia, length of stay in hospital (OR 1.034, 95% CI 1.004-1.065, p= 0.024); and for cough, pulmonary involvement >50% during COVID-19 infection (OR 3.793, 95% CI 1.184-12.147, p= 0.025) were found to be independent risk factors. ROC curves are shown in Figure 3 ( 13 ). No significant results were found for any other PASC.

**Table 1:** Demographic characteristics and clinical findings of patients, n= 256

Age	52.88 ± 16.43
Gender (n, %)	
Male	121 (47.27)
Female	135 (52.73)
Chronic lung disease (n, %)	
Chronic obstructive pulmonary disease	12 (4.94)
Asthma	19 (7.82)
Other	9 (3.52)
Other comorbidities (n, %)	141 (56.63)
Diabetes mellitus	44 (17.67)
Hypertension	81 (32.53)
Chronic cardiac disease	37 (14.86)
Chronic renal disease	15 (6.02)
Thyroid disease	16 (6.4)
Malignancy	16 (6.43)
Extent of lung involvement at the time of COVID-19 diagnosis (n, %)	
No lung involvement	61 (23.82)
<50% of the lung	149 (59.84)
>50% of the lung	39 (15.66)
Symptoms in the course of COVID-19 (n, %)	
Cough	137 (53.73)
Sputum	28 (10.98)
Dyspnea	111 (43.53)
Chest pain	22 (8.63)
Fever	36 (37.88)
Hemoptysis	3 (1.18)
Presence of hypoxia (SpO2 <%92) (n, %)	101 (39.61)
Presence of cytokine storm (n, %)	36 (14.23)
Anti-inflammatory drug use (n, %)	
Tocilizumab	28 (71.79)
Anakinra	7 (17.95)
Pulse steroid	3 (7.69)
Tocilizumab + anakinra	1 (2.56)
Treatment for COVID-19 (including favipiravir, hydroxychloroquine, or azithromycin) (n, %)	
No	37 (14.57)
Yes	217 (85.43)
Favipravir + hydroxychloroquine	179 (70.2)
Anti-coagulant	191 (74.9)
Steroid	107 (41.96)
Other	143 (55.86)
Treatment planned at discharge (n, %)	
Low molecular weight heparin	88 (36.82)
Steroid	33 (13.47)
Long-term oxygen therapy	22 (8.63)

**Table 2:** Ongoing symptoms according to frequency and duration in the course of long COVID

Symptoms	n (%)	Duration (day, min-max)
Dyspnea	106 (42.40)	90 (7-300)
Cough	33 (13.15)	60 (10-360)
Fatigue	71 (28.29)	60 (10-300)
Arthralgia-myalgia	41 (16.33)	60 (2-270)
Chest pain	12 (4.78)	60 (14-100)
Back pain	18 (7.17)	90 (15-180)
Palpitation	16 (6.37)	105 (7-270)
Headache	16 (6.37)	105 (4-180)
Difficulty concentrating-forgetfulness	14 (5.58)	80 (28-270)
Gastrointestinal symptoms (nausea-vomiting-diarrhea)	12 (4.78)	70 (2-270)
Dizziness	9 (3.59)	100 (4-240)
Skin lesion	9 (3.59)	35 (7-300)
Loss of taste	9 (3.59)	30 (10-240)
Loss of smell	9 (3.59)	35 (10-120)
Sputum	7 (2.8)	35 (10-120)
Wheezing	3 (1.2)	60 (90-100)
Runny nose	1 (0.4)	60*
Sore throat	1 (0.4)	90*
Dry throat	1 (0.4)	150*
Backache	4 (1.59)	65 (30-270)
Vision loss	1 (0.4)	210*
Tinnitus	1 (0.4)	120*
Chills	4 (1.59)	75 (5-150)
Numbness in extremities	6 (2.39)	90 (10-180)
Hair loss	5 (1.99)	60 (60-80)
Anorexia	3 (1.2)	10; 100*
Sweating	9 (3.59)	90 (30-150)
Nightmares	1 (0.4)	60*
Hearing loss	3 (1.2)	150; 180*

*Due to the limited number of valid observations within the relevant variables, the values of the observations are presented in lieu of descriptive statistics.

## DISCUSSION

### Prevalence of Long COVID, Distribution, and Duration of Symptoms

Long COVID is a multisystem disease with varying prevalence and symptom duration observed across studies conducted in different countries. In a follow-up study in Wuhan, Huang C et al. have shown that 76% of the 1.733 participants still suffered from at least one symptom six months after the onset of COVID-19 ( 15 ). Chen et al. reported that the global prevalence of long COVID was about 43%. Among patients who needed hospitalization, the prevalence increased to 57%. The researchers found the hospitalization rate to be 49% among women and 37% among men. The rate also varied by location, with the highest reported in Asia at 49%. Europe and North America followed at 44% and 30%, respectively ( 14 ). A systematic review reported persistent symptoms at 3-6 months in a median of 57% of hospitalized and 26% of non-hospitalized patients ( 16 ). In the Turkish Thoracic Society-TURCOVID multicenter registry cohort, comprising 504 patients, the prevalence of long COVID was recorded as 57.9%. Furthermore, one year after the acute infection, the prevalence persisted at 27.1% ( 17 ). In our study, which included 1.050 hospitalized patients from April 2020 to January 2021, a total of 256 individuals reported one or more long COVID symptoms. Consequently, the prevalence of long COVID in our study cohort was determined to be 24.3%.

While a majority of cases exhibit a self-limited course, with symptoms resolving or improving within 3-6 months, a notable observation from our assessment of long COVID symptoms is that approximately 10% to 30% of COVID-19 patients will continue to experience one or more chronic symptoms. Additionally, it has been reported that long COVID is more prevalent among young female patients who initially experienced mild illness ( 18 , 19 , 20 ). In our study, of the 256 patients who reported at least one PASC after acute infection, the median age was 52.8, and 52.7% were female, compatible with the literature.

The clinical spectrum of long COVID comprises a wide range of symptoms. Huang C. et al. reported that fatigue or muscle weakness and sleep difficulties were the most common symptoms in 63% and 26% of the patients, respectively ( 15 ). The most commonly reported non-neurological, persistent symptoms in different studies included fatigue or muscle weakness, joint pain, chest pain, palpitations, shortness of breath, and cough ( 17 , 21 , 22 , 23 , 24 ). Sarıoğlu et al. evaluated the clinical and radiological outcomes of long COVID in 126 patients on the third month after discharge and identified the most common persisting symptoms as shortness of breath (32.5%), cough (12.7%), and muscle pain (12.7%) ( 25 ). In our study, the five most frequently observed PASC symptoms, both in terms of patient numbers and symptom duration eight weeks after discharge, were dyspnea, fatigue, arthralgia-myalgia, cough, and back pain, respectively.

### Risk Factors for PASC Development

According to a study from the literature, the factors predicting the risk of developing PASC at the time of COVID-19 diagnosis include type 2 diabetes, circulating SARS-CoV-2 viremia, EBV reactivation, and specific autoantibodies such as type 1 interferon ( 26 ). Except for women, increasing age, having asthma, and being overweight or obese were also associated with an increased risk of long COVID ( 27 ). In our study, when the comorbidities of the 256 patients were evaluated, it was found that nearly half had at least one comorbidity. 16.28% of all patients had chronic pulmonary disease, and in the multivariate analysis, we found no statistically significant relationship between any long COVID-related symptom and age or gender.

Lopez-Leon et al. suggest that women and individuals aged between 40 and 54 years are more susceptible to experiencing long COVID ( 28 ). Moreover, they propose that the severity of the acute illness correlates with the number of symptoms emerging after the infection has resolved. On the other hand, Townsend et al. reported that more than half of the patients had persistent fatigue at a median of 10 weeks after initial symptoms first appeared, and there was no association between illness severity and fatigue, suggesting that long COVID may manifest irrespective of the initial disease severity ( 29 ). Jacobson K.B. et al. found substantial, persistent symptoms and functional impairment even in non-hospitalized patients, similar to patients with severe COVID-19 ( 30 ). Our study found pulmonary involvement of >50% during COVID-19 infection to be an independent risk factor for post-discharge cough as a PASC. Also, we found a statistically significant correlation between lung involvement during COVID-19 infection and dizziness. When examining the risk factors associated with the persistence of dyspnea eight weeks after the diagnosis of acute COVID-19 infection, significant associations were observed with known lung disease (including the presence of COPD), a history of intensive care support during the acute infection, the requirement for long-term oxygen therapy upon discharge, and the occurrence of a cytokine storm during hospitalization. Among them, having lung disease was an independent risk factor for dyspnea as a PASC. Our study found no correlation between any other PASC (such as chest pain, backache, headache, myalgia, fatigue, or loss of taste) and the severity of acute COVID-19 infection.

Sahin et al. assessed the olfactory and gustatory functions in COVID-19 patients, revealing that olfactory and gustatory dysfunctions were more prevalent in patients who exhibited clinical symptoms ( 31 ). Additionally, they determined that the median duration of olfactory dysfunction was seven days (range= 0-30), with one patient experiencing prolonged olfactory dysfunction persisting for 45 days after diagnosis. In our study, we observed a longer median duration of loss of smell, which was 35 days (range= 10-120). Additionally, we identified a significant correlation between the loss of smell and the length of hospital stay during the COVID-19 infection. Asadi-Pooya AA et al. demonstrated in their research that presentation with respiratory problems at the onset of illness was significantly associated (Odds ratio= 1.425; 95% confidence interval= 1.177-1.724; p= 0.0001), and a shorter length of hospital stay was inversely associated with long COVID syndrome (Odds ratio= 0.953; 95% confidence interval= 0.941-0.965; p= 0.0001). Likewise, employing logistic regression models, we identified a correlation between the hospitalization duration and long COVID. Consequently, through multivariate analyses, it emerged as an independent risk factor for myalgia as a PASC ( 32 ).

### Limitations

A primary limitation of our study arises from the fact that not all inpatients from the study period could be reached, as the study was conducted through a telephone survey. Additionally, the body mass index (BMI), which constitutes a confounding factor and is known to correlate with long COVID, could not be assessed due to the inherent constraints of this methodology ( 27 ). Another important limitation of this study is that it did not include a comparison between the two groups of patients, i.e. those exhibiting long COVID-related symptoms and those without. We conducted a descriptive analysis for all patients with at least one long COVID-related symptom as a result of the questionnaire we applied. We did not evaluate the patients who did not have any PASC. The study was conducted prior to the commencement of COVID-19 vaccine administration in our country. As a result, the impact of vaccines on PASC could not be assessed either. Several recent studies on immunophenotyping in the literature provide insights into the immunological aspects of long COVID ( 33 , 34 , 35 ). Recent literature has emerged concerning a specific innate immune cell type, NK cells, which play a crucial role in both COVID and potentially in the context of long COVID. Particularly, two distinct NK cell phenotypes (adaptive NK phenotype and NK cell with unique early IFNa signatures) are closely linked to the severity of COVID-19 ( 36 , 37 ). Unfortunately, our study was designed retrospectively, involving the examination of hospital records of patients exhibiting at least one persistent long COVID symptom. No serum samples were taken from the patients. Therefore, it was impossible to perform immunological phenotyping in our study. However, these aspects will serve as future directions for further evaluating our long COVID cohort, aiming to investigate the potential associations of these novel phenotypes with the condition.

## CONCLUSION

We performed univariate analyses for each PASC to evaluate the relationship between the demographic characteristics and the clinical findings. We found dyspnea, fatigue, arthralgia-myalgia, cough, and back pain to be our study’s five most common long COVID symptoms. The main risk factors for cough and dizziness as PASC were the severity of lung involvement during COVID-19 infection; on the other hand, the risk factors for persistence of dyspnea were having lung disease, history of ICU support and presence of cytokine storm during acute infection, and long-term oxygen therapy requirement at discharge. Among them, having lung disease was an independent risk factor for dyspnea as a PASC. Moreover, based on the outcomes of multivariate analyses, the duration of hospitalization emerged as an independent risk factor for myalgia, while pulmonary involvement exceeding 50% during the initial COVID-19 infection stood out as an independent risk factor for persistent cough in the context of PASC. These findings lead to the conclusion that long COVID is a multisystem disease developed regardless of the severity of acute COVID-19 infection, and its management requires a multidisciplinary approach. The underlying pathophysiology of this condition remains unclear, and studies focusing on this topic exhibit heterogeneity, encompassing varying levels of patient severity and differing time frame analyses. COVID-19 survivors will require significant healthcare services due to their prolonged symptoms. We hope that our findings will contribute to a better understanding of the implications of PASC and provide valuable guidance for optimal clinical management, particularly for policymakers and stakeholders in our country.

## Ethical Committee Approval

This study was approved by Gazi University Ethics Committee (Decision no: E-77082166-604.01.02-80610, Date: 26.04.2021).

## CONFLICT of INTEREST

The authors declare that they have no conflict of interest.

## AUTHORSHIP CONTRIBUTIONS

Concept/Design: YY, İKO, MD, BÖŞ, MCT, ŞD, BÇ, NK, TUÇ, NYD

Analysis/Interpretation: YY, BÖŞ, İKO, MD

Data acqusition: All of authors

Writing: YY, BÖŞ, MCT, ŞD, BÇ

Clinical Revision: YY, BÖŞ, MD, İKO

Final Approval: All of authors
